# A pyroptosis-related gene signature provides an alternative for predicting the prognosis of patients with hepatocellular carcinoma

**DOI:** 10.1186/s12920-023-01431-z

**Published:** 2023-01-07

**Authors:** Dezhao Lin, Zhuoyan Chen, Yuan Zeng, Yinrong Ding, Luying Zhao, Qian Xu, Fujun Yu, Xian Song, Xiaohong Zhu

**Affiliations:** 1 Department of Surgical Oncology, Wenzhou Hospital of Traditional Chinese Medicine Affiliated to Zhejiang Chinese Medicine University, Wenzhou, Zhejiang People’s Republic of China; 2 Department of Gastroenterology, The First Affiliated Hospital of Wenzhou Medical University, Nanbaixiang, Ouhai District, Wenzhou, Zhejiang People’s Republic of China; 3 Department of Anesthesiology, The First Affiliated Hospital of Wenzhou Medical University, Nanbaixiang, Ouhai District, Wenzhou, Zhejiang People’s Republic of China

**Keywords:** Hepatocellular carcinoma (HCC), Pyroptosis, Gene signature, Overall survival, Immune status, Drug sensitivity

## Abstract

**Background:**

Hepatocellular Carcinoma (HCC) is a common malignant neoplasm with limited treatment options and poor outcomes. Thus, there is an urgent need to find sensitive biomarkers for HCC.

**Methods:**

Gene expression and clinicopathological information were obtained from public databases, based on which a pyroptosis-related gene signature was constructed by the least absolute shrinkage and selection operator Cox regression. The applicability of the signature was evaluated via Kaplan–Meier curve and time-dependent ROC curve. TIMER, QUANTISEQ, MCPCOUNTER, EPIC, CIBERSORT, ssGSEA, and ESTIMATE were employed to assess the immune status. Comparisons between groups were analyzed with Wilcoxon test. Pearson and Spearman correlation analyses were adopted for linear correlation analysis. Genetic knockdown was conducted using siRNA transfection and the mRNA expression levels of interest genes were measured using quantitative reverse transcription PCR. Finally, protein levels in 10 paired tumor tissues and adjacent non-tumor tissues from HCC patients were measured using immunohistochemistry.

**Results:**

A pyroptosis-related gene signature was established successfully to calculate independent prognostic risk scores. It was found that survival outcomes varied significantly between different risk groups. In addition, an attenuated antitumor immune response was found in the high-risk group. Meanwhile, multiple immune checkpoints were up-regulated in high-risk score patients. Cell cycle-related genes, angiogenesis-related genes and tumor drug resistance genes were also markedly elevated. Knockdown of prognostic genes in the signature significantly inhibited the expression of immune checkpoint genes and angiogenesis-related genes. Besides, each prognostic gene was expressed at a higher level in HCC tissues than in adjacent normal tissues.

**Conclusions:**

We successfully established a novel pyroptosis-related gene signature which could help predict the overall survival and assess the immune status of HCC patients.

**Supplementary Information:**

The online version contains supplementary material available at 10.1186/s12920-023-01431-z.

## Introduction

Hepatocellular carcinoma (HCC) is one of the most frequently diagnosed malignant tumors, accounting for more than 90% of all liver cancer cases [1], and ranking fifth of the incidence and third in mortality of all malignancies worldwide [2]. Risk factors for HCC include chronic hepatitis B and C virus infections, alcohol abuse, nonalcoholic fatty liver disease, and exposure to dietary toxins such as aflatoxins [3]. Patients are often diagnosed with HCC at advanced stages with poor prognosis [1], and unclear molecular mechanisms lead to the poor understanding of HCC prognosis. The overall survival (OS) of HCC varies across the world, with a 5-year survival rate of 18% in the United States, and 12% in China [4]. Therefore, it is primarily important to clarify the molecular mechanisms underlying the poor prognosis of HCC and explore new prognostic biomarkers for HCC.

Pyroptosis, an inflammatory form of cell death triggered by certain inflammasomes, is primarily mediated by caspase 1/4/5 (11)-cleaved GSDMD (gasdermin D). Afterwards, GSDMD-N oligomerizes form membrane pores, leading to the release of activated cytokines and pyroptotic cell death [5–7]. Some studies reported that pyroptosis played a double-edged role in cancer [8]. While still an ambiguous process in cancer, pyroptosis could not only play a potent and persuasive role to conquer apoptosis resistance but also a crucial role in immunity [9]. Conversely, pyroptosis can induce damage to healthy tissues and establish a microenvironment suitable for tumor growth and metastatic progression [10]. Obviously, pyroptosis involves complex and contentious processes, so that its role and detailed mechanism in oncogenesis deserves extensive exploration. Although the exact link between pyroptosis and HCC is presently unclear, increasing research has begun to focus on HCC and pyroptosis [11, 12].

In this study, we attempted to explore the association between pyroptosis and HCC by establishing a predictive signature based on pyroptosis-related genes strongly related to poor outcomes of HCC, hoping that our findings could provide a new signature that could predict the clinical survival outcome and help design individualized treatment for HCC patients.

## Methods

### Data acquisition

The data were obtained from public databases. HCC samples collected before 2011 were excluded because of the excessive length of storage. Finally, data of 209 HCC patients were obtained from The Cancer Genome Atlas hepatocellular carcinoma (TCGA-LIHC) portal (https://portal.gdc.cancer. gov/repository). Additional 231 tumor samples and 199 adjacent normal tissue samples were collected from the International Cancer Genome Consortium hepatocellular carcinoma (ICGC-LIRI-JP) portal (https://dcc.icgc.org/projects/LIRI-JP). Also, an independent cohort containing 10 paired HCC samples was recruited. Clinical data for these patients are presented in Table 1. The present study follows access policies and publication guidelines. Then, 69 pyroptosis-related genes were obtained from the previous literature [8, 13–17] (Additional file 1: Table S1).

**Table 1:** Clinical characteristics of the HCC patients used in this study

	TCGA-LIHC cohort	ICGC-LIRP-JI cohort
*No. of patients*	209	231
*Age (median, range)*	59 (16–90)	67 (31–89)
*Gender*		
Female	65 (31.1%)	61 (26.4%)
Male	144 (68.9%)	170 (73.6%)
*Grade*		
Grade 1	35 (16.7%)	NA
Grade 2	103 (49.3%)	NA
Grade 3	62 (29.7%)	NA
Grade 4	7(3.3%)	NA
Unknown	2 (1.0%)	NA
*Stage*		
I	91 (43.5%)	36 (15.6%)
II	54 (25.8%)	105 (45.5%)
III	49 (23.4%)	71 (30.7%)
IV	1 (0.5%)	19 (8.2%)
Unknown	14 (6.7%)	0 (0%)
*Survival status*		
Alive	159 (76.1%)	189 (81.8%)
Deceased	50 (23.9%)	42 (18.2%)
*Follow up (median, range)*	588 (1–1363)	900 (10–2160)

### Identification of prognostic genes

The differentially expressed genes (DEGs) between HCC and adjacent non-tumorous tissues were screened with an FDR < 0.05 and log2 (fold-change) > 0 from the whole genes for candidate genes by the "limma" R package. Univariate Cox analysis was implemented to identify significant prognostic genes from the DEGs (*P* < 0.05). Correlation networks were performed through “igraph” and “reshape2” packages. Protein–protein interaction (PPI) networks were constructed with the STRING database.

### Gene signature establishment and validation

When constructing the prognostic gene model, the collected data were processed based on the least absolute shrinkage and selection operator (LASSO) penalized Cox regression analysis, and the penalty parameters were obtained by cross validation with the R package "glmnet" tool. When the dataset was large, K-Fold Cross Validation analysis was used, so that the optimal model and parameters can be selected for function evaluation. When the dataset was small (N < 50), the leave-one-out Cross Validation analysis was used because it uses more training samples in each iteration. The LASSO analysis was mainly used to select variables and determine the model that met the interpretation requirements according to the regression coefficient. The standardized expression matrix of candidate prognostic genes was the independent variable in the regression equation, and the dependent variables were OS and patient status. Based on the analysis of the expression value of each gene and the corresponding regression coefficient, the risk score of the patients was determined: score = esum (expression of each gene × Correspondence coefficient). All patients were divided into high- and low-risk groups based on the median score. The "Rtsne" R software was used for principal component analysis to determine the distribution of each group. The survival status of patients was analyzed based on the Kaplan–Meier test. The ROC curve was drawn by processing the relevant data, and then the AUC value was calculated by the R package “survivalROC” so as to determine the sensitivity of variables.

### Identification of independent prognostic factors for OS in HCC

To evaluate the predictive power of the risk score, each prognostic gene in the signature (CASP3, IRAK1, MAPK1, MAPK3 and YWHAB) and the clinical risk factors, including age (< 60 vs. ≥ 60), gender (male vs. female), tumor grade (G1/2 vs. G3/4), tumor stage (I/II vs. III/IV), univariate and multivariate Cox regression analyses were performed to identify independent prognostic factors.

### Immune status in distinct risk groups

Five algorithms (TIMER, QUANTISEQ, MCPCOUNTER, EPIC and CIBERSORT) were applied to evaluate the correlation between immune cells and risk scores by “immunedeconv” R package [18]. The differences between these five algorithms are shown in Additional file 2: Table S2. Single-sample gene set enrichment analysis (ssGSEA), ESTIMATE and CIBERSORT were performed to assess the immune status in high- and low-risk groups by the "GSVA", “estimate” and “limma” R package based on all of the expressed genes. The expression levels of immune checkpoint genes in different risk groups were analyzed by Wilcoxon test.

### Gene ontology (GO) and Kyoto Encyclopedia of Genes and Genomes (KEGG) pathway analyses

To detect the potential biological functions and pathways in different risk groups, GO and KEGG pathway analyses were carried out using the “clusterProfiler” and “enrichplot” packages based on all of the expressed genes [19–21].

### Cell cycle and angiogenesis-related genes expression in different risk groups

The expression of cell cycle and angiogenesis-related genes in different risk groups was compared using the Wilcoxon test.

### Tumor drug resistance genes and chemotherapy sensitivity analysis

The NCI-60 database containing 60 different cancer cell lines from 9 different types of tumors was accessed through the CellMiner interface (https://discover.nci.nih.gov/cellminer/). Altogether 263 drugs on clinical trials or approved by the US Food and Drug Administration, were used to evaluate the correlation between prognostic gene expression and drug sensitivity, and that between the prognostic model and tumor drug resistance genes by Pearson and Spearman correlation analyses respectively.

### Cell lines, cell culture and cell transfection

HCC cell line Huh7 was purchased from the Cell Bank of the Chinese Academy of Sciences (Shanghai, China). To reduce the risk of microbial contamination, cells were cultured in a suitable medium containing 10% fetal bovine serum (FBS; Gibco) and supplements of penicillin (100 U/mL) and streptomycin (100 g/mL) in a humidified environment with 5% CO2/95% air at 37 °C. Gene silencing was achieved using siRNA. siRNA was transfected into cells using Lipofectamine 3000 (Life Technologies), according to the manufacturer’s instructions. Transfected cells were analyzed 48 h after transfection with siRNA. siRNA sequences are listed in Additional file 3: Table S3.

### Quantitative reverse transcription PCR (qRT-PCR)

Total RNA was extracted from the cells using TRIzol reagent (Thermo Scientific, Cat# 15596018). Then, the RNA was quantified using Nanodrop. The RNA was reverse-transcribed into DNA using a Prime Script RT Reagent Kit (Takara, Cat#RR036A) following the manufacturer’s instructions. Isolated DNA was subjected to qRT-PCR analysis using the CellAmp™ Direct TB Green® RT-qPCR Kit (Takara, Cat# 3735A). Primer sequences are listed in Additional file 4: Table S4.

### Immunohistochemistry (IHC)

Ten pairs of HCC and adjacent non-tumorous tissues collected by the First Affiliated Hospital of Wenzhou Medical University (Wenzhou, China) were selected as samples for IHC detection, so as to determine the protein expression of prognostic genes in these samples and evaluate the prognostic value of these genes. The research content was approved by the ethics review committee of the university, and all the subjects were informed and agreed. The samples were collected and then fixed in 10% formalin, placed at room temperature for half an hour, subsequently embedded in paraffin, processed into 4-μm slices, deparaffinized, rehydrated, and boiled for 10 min. Then immerse the slice in 3% hydrogen peroxide solution for 10 min, which could achieve the purpose of inactivation and avoid non-specific binding. Incubate the slice with 1% FBS in PBS for half an hour. After incubation, the sections were stained by the secondary antibody combined with the primary antibody and HRP. The antibodies are detailed in Additional file 5: Table S5. Then the sections were stained continuously with 3,3′-diaminobenzidine and hematoxylin. After dehydration, the samples were sealed, observed and photographed. Representative pictures of prognostic genes were displayed in our study. Finally, a quantitative analysis of IHC staining was conducted by ImageJ software.

### Statistical analyses

Group comparison was made by the Wilcoxon test. Chi-square test was used to check the difference of results of categorical variables, and Kaplan Meier test was used for the difference of survival rates between the two groups. Cox regression analysis was conducted on the collected data to determine the factors closely related to OS, and the corresponding regression analysis model was established based on the results. The relationship between risk scores and drug resistance genes was determined by the Spearman method. The relationship between prognostic gene expression and drug sensitivity was judged by the Pearson coefficient. R4.0.2 and SPSS23.0 software were used for data statistical analysis, and the correlation curves were drawn. *P* < 0.05 indicates that the difference of results is statistically significant.

## Results

### Screening of pyroptosis-related prognostic genes

**Fig. 1:**
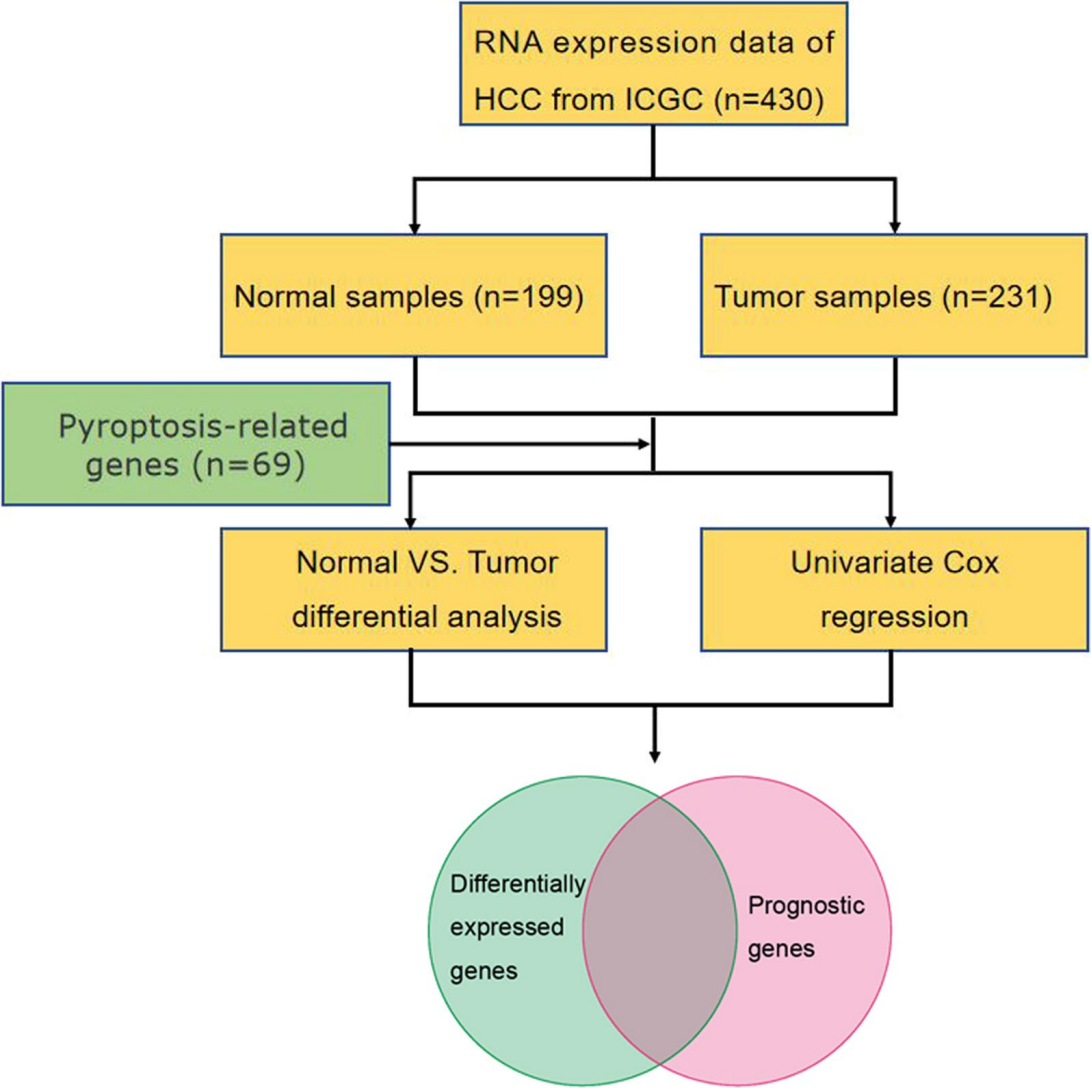
Flowchart of the identification of candidate genes. The whole gene expression data set was subset to putative pyroptosis-related genes, and then differentially expressed genes (DEGs) from differential analysis were intersected with prognostic genes from univariate cox regression

**Fig. 2:**
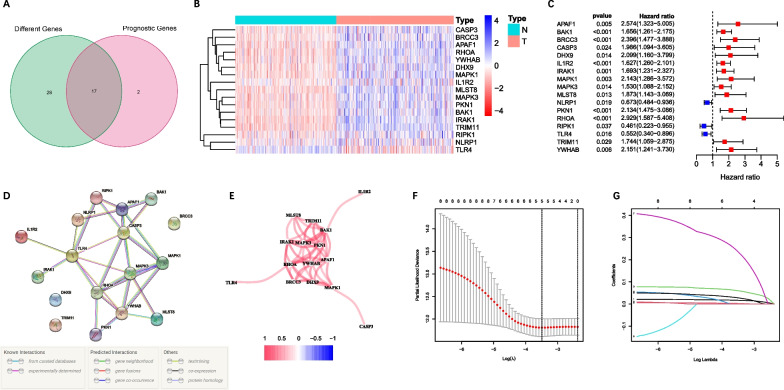
Screening pyroptosis-related prognostic genes and constructing a prognostic signature model. **A** Venn diagram of DEGs and prognostic genes. **B** The heatmap of candidate genes expression. **C** Forest plots showed the univariate Cox regression analysis of the candidate genes. **D** The PPI network of the candidate genes. **E** Gene correlation networks of candidate genes. Red color means a positive correlation, blue color means a negative correlation. **F** Selection of the penalty parameter (λ) in the LASSO model by tenfold cross-validation. The lower x-coordinate is the value of Log(λ), the upper x-coordinate is the number of candidate genes, and the dashed line is the number of candidate genes corresponding to the cutoff value of Log(λ). **G** LASSO coefficient profiles of the expression of 21 candidate genes

The flowchart for the screening of candidate genes was exhibited in Fig. 1. Among the 69 pyroptosis-related genes, 45 of them were expressed differentially between tumor and adjacent non-tumorous tissues, and 19 of them were linked to OS (Fig. 2A). Ultimately, 17 genes were survival-related and discovered to have significant differential expression, based on which a pyroptosis-related gene signature was constructed. Figure 2B–C shows the prognostic genes and their hazard ratio. PPI networks and gene correlation networks revealed the interactions among these prognostic genes (Fig. 2D–E).

### Construction of the prognostic signature

**Fig. 3:**
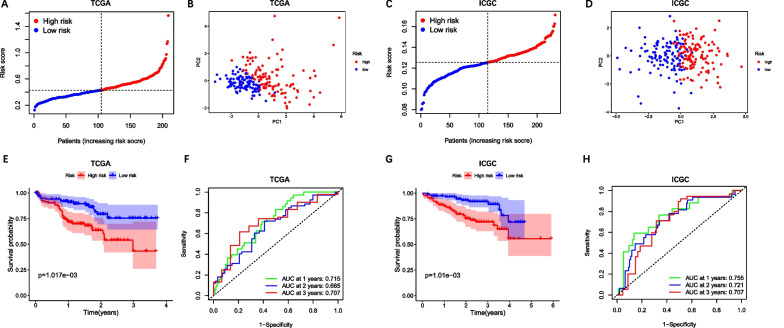
Prognostic analysis of the pyroptosis-related signature in TCGA and ICGC cohorts. TCGA cohort (**A**, **B**, **E**, **F**), ICGC cohort (**C**, **D**, **G**, **H**). **A**, **C** Distribution of the risk scores. **B**, **D** PCA analysis shows the distribution of different risk groups. **E**, **G** Kaplan–Meier curves for OS of patients in high- and low-risk groups. **F**, **H** Time-dependent ROC curves of 1-year, 2-year and 3-year OS

Five genes with maximum prognostic value (CASP3, IRAK1, MAPK1, MAPK3 and YWHAB) were found using LASSO Cox regression analysis (Fig. 2F–G). A pyroptosis-related prognostic model was constructed using the genes mentioned above in the TCGA cohort. The risk score = 0.97 × 10^–2^ × expression level of CASP3 + 0.65 × 10^–2^ × expression level of IRAK1 + 0.53 × 10^–2^ × expression level of MAPK1 + 0.83 × 10^–2^ × expression level of MAPK3 + 0.18 × 10^–2^ × expression level of YWHAB. According to the median cut-off value, the patients were grouped into either a high-risk group or a low-risk group (Fig. 3A). It was clearly demonstrated that patients in distinct risk groups were separated in discrete directions (Fig. 3B). In addition, patient survival in high-risk score group was significantly poorer than that in low-risk score group (Fig. 3E). Besides, time-dependent ROC analysis showed excellent performance of the prognostic signature (TCGA: 1-year AUC = 0.716, 2-year AUC = 0.665, 3-year OS = 0.707) (Fig. 3F).

### Validation of the prognostic signature

We further examined the prognostic signature in the ICGC cohort for additional independent validation. Patients were separated into different risk groups using the same formula and median value from the TCGA cohort (Fig. 3C). The results in the ICGC cohort were virtually consistent with those in the TCGA cohort. Patients in different risk groups were separated in two directions and OS of patients in the high-risk group was significantly worse than that in the low-risk group. (Fig. 3D, G). The AUC of the pyroptosis-related signature for 1-, 2-, and 3-year OS were 0.755, 0.721 and 0.707, respectively (Fig. 3H).

### Clinical characteristics of patients in different risk groups

**Fig. 4:**
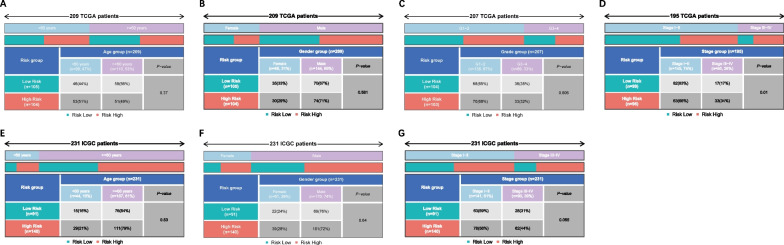
Clinical characteristics in different risk groups. **A**, **E** Age-stratified patients (< 60 year and ≥ 60 year) in high- and low-risk groups. **B**, **F** Gender-stratified patients (male and female) in high- and low-risk groups. **C** Patients at different grades (G1-2 and G3-4) in high- and low-risk groups. **D**, **G** Patients at different stages (stage I-II and stage III-IV) in high- and low-risk groups

The proportion of tumor stage I-II patients in the high-risk score group was significantly higher than that in the low-risk score group (*P* < 0.05), and vice versa for the proportion of tumor stage III-IV (Fig. 4D). Likewise, a similar trend was seen in the ICGC cohort (Fig. 4G), though the difference was not statistically significant (*P* = 0.055). In addition, no significant difference in age, gender and tumor grade was observed between the two groups (Fig. 4A–C, E–F). All these findings suggest that the tumor stage tended to be higher in patients with high-risk scores.

### Independent prognostic analysis of the pyroptosis-related signature

**Fig. 5:**
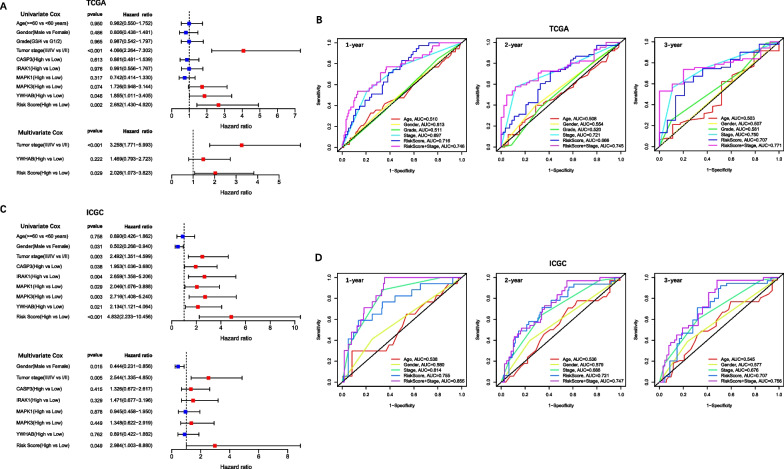
Univariate and multivariate Cox regression analyses and time-ROC curves of clinical characteristics with risk scores. TCGA cohort (**A**, **B**), ICGC cohort (**C**, **D**). **A**, **C** Univariate and multivariate Cox regression analyses of clinicopathological parameters (age, gender, tumor grade, tumor stage), each prognostic gene in the signature (CASP3, IRAK1, MAPK1, MAPK3 and YWHAB) and the risk score. **B**, **D** The time-ROC curves of clinical characteristics, risk score, and risk score combined with tumor stage

The independent prognostic value of the risk score, each prognostic gene in the signature and clinical features was evaluated by univariate and multivariate Cox analyses. Tumor stages, YWHAB and risk scores in the TCGA cohort and gender, tumor stages, CASP3, IRAK1, MAPK1, MAPK3, YWHAB and risk scores in the ICGC cohort significantly correlated with the patient survival prognosis in univariate analysis were subjected to multivariate analysis, and the results showed that the risk score and the tumor stage were statistically significant (*P* < 0.05) (Fig. 5A, C). Clearly, the risk score was a prominent risk predictor after adjustment for known clinical and pathologic factors, but no single gene showed an independent prognostic value. Furthermore, the combination of the risk score with the tumor stage could provide a more accurate prediction of 1-, 2-, 3- year OS in HCC (TCGA: 1-year AUC = 0.746, 2-year AUC = 0.745, 3-year OS = 0.771; ICGC: 1-year AUC = 0.855, 2-year AUC = 0.747, 3-year OS = 0.756) (Fig. 5B, D).

### Association between the signature and immune status

Next, five algorithms were performed to evaluate the correlation between immune cells and risk scores. It was found that the risk score was positively correlated with the infiltration of macrophages, myeloid dendritic cells, T cell regulatory (Treg), macrophage/monocytes and cancer-associated fibroblasts (Additional file 6: Fig. S1A, C). The expression of immune checkpoint genes including PDCD1 (PD-1), PDCD1LG2 (PD-L2), CTLA4, CD80, CD86, HAVCR2, LGALS9, CD276 and VTCN1 were also up‐regulated in the high‐risk group (Additional file 6: Fig. S1B, D). To further explore the immune status in different risk groups, we performed ssGSEA algorithm. In both the TCGA and the ICGC cohorts, the immune cell subpopulations including aDCs, iDCs, macrophages, Tfh as well as Treg showed high infiltration in the high-risk group (*P* < 0.05) (Additional file 6: Fig. S2A, B). Moreover, the scores of immune-related functions (CCR, Check point, MHC class I and parainflammation) in the high-risk group were significantly higher than those in the low-risk group (Additional file 6: Fig. S2C, D). Besides, CIBERSORT and ESTIMATE were also performed to distinguish immune status in high- and low-risk groups (Additional file 6: Fig. S2E–H).

### Function enrichment analysis

GO terms, such as cell cycle checkpoint, cell cycle G2/M phase transition, cell cycle G1/S phase transition, cell cycle DNA replication, signal transduction involved in cell cycle checkpoint and cellular response to hypoxia, were significantly enriched by GO enrichment analysis (Additional file 6: Fig. S3A, C). In addition, KEGG pathway terms including cell cycle, hepatitis B, PD-L1 expression and PD-1 checkpoint pathway in cancer, T cell receptor signaling pathway, VEGF signaling pathway, HIF-1 signaling pathway, EGFR tyrosine kinase inhibitor resistance and hepatocellular carcinoma were significantly enriched by KEGG analysis (Additional file 6: Fig. S3B, D). Intriguingly, the KEGG HCC signaling pathway was significantly enriched, whether in the TCGA database or the ICGC database.

### Expression levels of genes related to cell cycle and tumor angiogenesis

Wilcoxon tests showed that the expressions of cell cycle (CCNA2, CCNB1, CCNB2, CCND2, CCND3, CDC20, CDC23, CDC25A, CDC25B, CDC25C, CDK1, CDK2, CDK4, CDK7, CHEK1, CHEK2, E2F1, E2F3, E2F4 and GSK3B) and angiogenesis-related genes (HIF1A, FDGFRA, FDGFRB, FDGFA, FDGFB, NRP1, NRP2, ANGPT2, VEGFB, FGFR1, FGFR2, FGFR3, RCBO1, RCBC3, SLIT1 and SLIT2) were increased in the high-risk group than those in the low-risk group (Additional file 6: Fig. S4).

### Tumor drug resistance analysis

So far, tumor drug resistance remains a main problem in tumor therapy, which urged us to further explore the correlation between the risk score and tumor drug resistance. It was found in our study that the expression levels of MRP1, MRP4 and MRP5 in the high-risk group were higher than those in the low-risk group. Furthermore, the expression levels of MRP1, MRP4 and MRP5 were positively correlated with the risk score (Additional file 6: Fig. S5A–B). Besides, Pearson correlation analysis revealed an inverse correlation (*P* < 0.05) between the expression levels of the prognostic genes and chemosensitivity (Additional file 6: Fig. S5C).

### Knockdown of prognostic genes in HCC cell lines

To further substantiate the functions of the prognostic genes in the signature, siRNA knock-down experiments were performed. siRNA was transfected into HCC cells to inhibit the expression of CASP3, IRAK1, MAPK1, MAPK3, and YWHAB, respectively. Subsequently, we examined the changes in mRNA expression of immune checkpoint genes and angiogenesis-related genes. qRT-PCR results showed that the majority of immune checkpoint genes (PDCD1, HAVCR2, LGALS9 and VTCN1) and angiogenesis-related genes (PDGFRA, PDGFB, VEGFB and FGFR1) had significantly lower levels of mRNA expression in the GMPS knockdown group than in the control group (Additional file 6: Fig. S6).

### IHC

The protein expression of the target genes was determined by IHC. The results showed that the expression of CASP3, IRAK1, MAPK1, MAPK3 and YWHAB in HCC tissues was significantly higher than that in adjacent non-tumorous tissues, which is consistent with RNA-sequencing data from the public databases (Additional file 6: Fig. S7).

## Discussion

Despite considerable advances toward the understanding of the molecular mechanism of HCC [22–24], it remains a major public health problem, especially in China [25, 26]. The existing prognostic staging system still has many limitations in accurate prognosis prediction and individualized precision therapy [27, 28]. Therefore, continued efforts are needed to find better prognostic signatures to guide individualized treatment so that patients can profit more from precision therapy.

In this study, we built a pyroptosis-related gene signature, knowing that patients with similar clinical characteristics such as the tumor stage may have different outcomes due to the heterogeneity of different epigenetic and genetic backgrounds in tumor subtypes [29]. Our study identified that the risk score was an independent prognostic factor of OS and it was superior to the tumor stage in predicting the OS of HCC patients. Combined with the tumor stage, the prognostic signature performed better in predicting OS. Therefore, the combined use of the five-gene signature and tumor stage may be more conducive to predicting the prognosis of HCC.

CASP3 used to be assumed as an executioner of apoptosis. However, the latest viewpoint proposes that caspase-3 can cause GSDME-mediated pyroptosis [30, 31]. Also, it can promote cancer cell growth, cellular migration, invasiveness, and tumor angiogenesis [32–35]. IRAK1 is a critical mediator of toll-like receptor and interleukin-1 (IL-1) signaling pathways, playing a crucial role in innate immunity and inflammation. Disruption of these pathways is associated with numerous diseases, including malignancies including HCC [36, 37]. MAPK1 (also known as ERK2) and MAPK3 (also known as ERK1) belong to the MAP kinase family. Both of them are associated with the development and progression of multiple tumors including HCC [38–40]. YWHAB encodes a number of 14–3-3 family proteins, of which 14–3-3β regulates multiple signaling pathways in normal and cancer cells [41]. Up-regulation of the 14–3-3β enhances HCC cell migration and proliferation [42, 43]. Collectively, all these prognostic genes in the model have been reported to be involved in cancer initiation and progression and most of them show a close link with HCC, suggesting that these genes can potentially be used as prognostic biomarkers for HCC. Nevertheless, whether these genes affect the prognosis of HCC remains to be elucidated.

Subsequently, GO and KEGG pathway analyses were used to identify the potential role of DEGs. These genes were primarily manifested in cancer-associated pathways, such as cell cycle [44], focal adhesion [45, 46], ras protein signal transduction [47], canonical wnt signaling pathway [48, 49], cellular response to hypoxia [50], Notch signaling pathway [51, 52], VEGF signaling pathway [53], AMPK signaling pathway [54], HIF-1 signaling pathway [55, 56], NF-kappa B signaling pathway [57], mTOR signaling pathway [58, 59], MAPK signaling pathway [60] and HCC. Of these, HCC is in line with our study subject. Based on these results, we speculated that these DEGs may contribute to the poor prognosis of HCC via activating the above biological pathways. Simultaneously, immune-related pathways, including T cell receptor signaling pathway, B cell receptor signaling pathway, regulation of T cell activation, regulation of T cell differentiation and antigen processing and presentation were significantly enriched, implying that immune dysfunction may also account for poor outcomes in HCC patients.

Additionally, we also noted that cell cycle-related pathways (cell cycle checkpoint, cell cycle G1/S phase transition, cell cycle DNA replication and cell cycle G2/M phase transition) and tumor angiogenesis-related pathways (HIF-1 signaling pathway, VEGF signaling pathway, mTOR signaling pathway and MAPK signaling pathway) were markedly enriched [53, 55, 61–64]. Aberrant cell cycle is known as a common feature of tumorigenesis [65]. Also, tumor angiogenesis is a pivotal step in tumor growth, invasion and migration [66]. So, we performed a further study on the cell cycle and tumor angiogenesis and found that cell cycle and angiogenesis genes were aberrantly up-regulated in the high-risk group. Furthermore, we discovered that the knockout of prognostic genes in the signature significantly reduced the expression of angiogenesis genes in HCC cells. Accordingly, high-risk scores are likely to be associated with cancer cell proliferation and angiogenesis through regulating the aforementioned genes and pathways. Meanwhile, inhibiting the expression of pyroptosis-related prognostic genes can suppress tumor angiogenesis, thereby improving the prognosis of high-risk populations.

The tumor microenvironment (TME) plays an important role in tumor progression and metastasis [67]. It was found in this study that the infiltration of macrophages and Treg in the high-risk group was significantly higher than that in the low-risk group and the risk scores were positively correlated with the infiltration of macrophages, Treg and cancer associated-fibroblasts, which are known as important components of TME [68, 69]. Macrophages as well as Treg are known to suppress anti-tumor immunity and facilitate tumor progression [69–72]. Cancer-associated fibroblasts have also been reported to contribute to cancer progression [73, 74]. Intriguingly, immune checkpoint molecules of PD-1, PD-L2, CTLA4, CD80, CD86, HAVCR2, LGALS9, CD276 and VTCN1 were up-regulated in the high-risk group. Moreover, our study demonstrated a significant decrease in the expression of PDCD1, HAVCR2, LGALS9 and VTCN1 after silencing the prognostic genes in the signature. It is known that immune checkpoint inhibitory molecules can facilitate immune escape of cancer cells [75]. Hence, together with the immune dysfunction discussed above, we conclude that induction of immunosuppressive microenvironment seems to be associated with an unfavorable prognosis of high-risk patients, and inhibiting the expression of pyroptosis-related prognostic genes may improve tumor immunosuppression.

Unexpectedly, we discovered that aDCs, iDCs and MHC class I were enriched in the high-risk group, and all of them are related to antigen presentation. Notably, antigen processing and presentation was enriched in the high-risk group suggesting the prognostic genes in our study may change the TME and immune status through affecting antigen presentation, but specific mechanisms warrant further investigation.

Additionally, PD1/PDL1 checkpoint attracted our special attention. To the best of our knowledge, tumor cells can mediate tumor immune escape by utilizing the PD1 / PDL1 checkpoint [76, 77]. Interestingly, recent studies suggested that pyroptosis-induced inflammation could activate anti-tumor immune responses and sensitize cancer cells to anti-PD-1 therapy [78, 79]. More importantly, KEGG analysis revealed that PD-L1 expression and PD-1 checkpoint pathway in cancer was enriched in the high-risk group. Thus, the synergistic effect of pyroptosis induction and PD-1 inhibitors might produce potent anti-tumor effects in patients with high-risk scores, though additional studies are required to verify our supposition.

HCC is extremely resistant to traditional chemotherapeutics, with only certain drugs yielding effective clinical responses [80]. Knowing that chemoresistance is a difficult problem in HCC treatment, we examined the impact of pyroptosis-related genes on chemosensitivity. Of note, the expression levels of target genes were inversely related to chemosensitivity and tumor drug resistance genes (MRP1, MRP4 and MRP5) were overexpressed in the high-risk group. Based on the aforementioned results, it is reasonable to believe that chemotherapy resistance in high-risk patients may be caused by up-regulated expression of MRP1, MRP4 and MRP5. In addition, target genes were significantly enriched in HIF-1 signaling pathway and cellular response to hypoxia in our study. Hypoxia is known to closely contribute to chemoresistance in cancers and HIF1A is an important target for hypoxia-driven drug resistance [81, 82]. Therefore, we speculate that hypoxia is one of the possible causes of chemoresistance in the high-risk group. Chemotherapy drugs can activate caspase 3, thereby specifically cleaving GSDME and ultimately inducing pyroptosis [31, 83]. Recently, a combined therapy consisting of DNA demethylation (promote GSDME expression) and chemotherapy (trigger caspase-3-involved pyroptosis of tumor cells) attracts our sight. This combination strategy could stimulate immune responses through pyroptosis-induced cytokine release and suppress tumor growth, metastasis, and recurrence [84]. It is clear that caspase-3 overexpression played a significant role in this process in the high-risk group, indicating that this combined therapy strategy can better improve the poor outcome in the high-risk group.

## Conclusion

In this study, we successfully generated a strong prognostic signature which we believe can help further refine the prognostic predictive power of HCC. This pyroptosis-related signature may provide new insights into the immunity of HCC and suggest a possible direction for individualized treatment of HCC in future.

## Supplementary Information

**Additional file 1.** The description of pyroptosis related genes.

**Additional file 2.** Five kinds of immune infiltration algorithm principles.

**Additional file 3.** The siRNA sequences used in this study.

**Additional file 4.** The primer sequences used in this study.

**Additional file 5.** Information of the antibody used in IHC.

**Additional file 6.** Figs. S1-S7.

## Data Availability

The datasets analysed during the current study are available in the TCGA repository (https://portal.gdc.cancer.gov/repository) and ICGC repository (https://dcc.icgc.org/projects/LIRI-JP). Additional data can be requested from the corresponding author.

## Abbreviations

HCC: Hepatocellular carcinoma
OS: Overall survival
GSDM: Gasdermin
TCGA: The Cancer Genome Atlas
ICGC: International Cancer Genome Consortium
LASSO: The least absolute shrinkage and selection operator
DEGs: Differentially expressed genes
ROC: Receiver operating characteristic
AUC: Area under the curve
GO: Gene Ontology
KEGG: Kyoto Encyclopedia of Genes and Genomes
GSEA: Gene set enrichment analysis
IHC: Immunohistochemistry
qRT-PCR: Quantitative reverse transcription PCR
Treg: T cell regulatory
TME: Tumor microenvironment
