# Supplementary material for: A pyroptosis-related gene signature provides an alternative for predicting the prognosis of patients with hepatocellular carcinoma
Source: BMC Med Genomics. 2023 Jan 7;16:2. doi: 10.1186/s12920-023-01431-z (PMC9826587; doi:10.1186/s12920-023-01431-z)
Supplement: Supplementary file 1 — Additional file 1. The description of pyroptosis related genes. [file 12920_2023_1431_MOESM1_ESM.docx]

Table S1. The description of [pyroptosis](javascript:;) related genes

| Gene Symbol | Gene Description |
| --- | --- |
| AIM2 | absent in melanoma 2 |
| APAF1 | apoptotic peptidase activating factor 1 |
| BAK1 | BCL2 antagonist/killer 1 |
| BAX | BCL2 associated X, apoptosis regulator |
| BRCC3 | BRCA1/BRCA2-containing complex subunit 3 |
| BTK | Bruton tyrosine kinase |
| CARD8 | caspase recruitment domain family member 8 |
| CASP1 | caspase 1 |
| CASP3 | caspase 3 |
| CASP4 | caspase 4 |
| CASP5 | caspase 5 |
| CDK9 | cyclin dependent kinase 9 |
| CMPK2 | cytidine/uridine monophosphate kinase 2 |
| CTSB | cathepsin B |
| DHX9 | DExH-box helicase 9 |
| ERN1 | endoplasmic reticulum to nucleus signaling 1 |
| FADD | Fas associated via death domain |
| GSDMA | gasdermin A |
| GSDMB | gasdermin B |
| GSDMC | gasdermin C |
| GSDMD | gasdermin D |
| HK1 | hexokinase 1 |
| IFI16 | interferon gamma inducible protein 16 |
| IFNAR1 | interferon alpha and beta receptor subunit 1 |
| IFNAR2 | interferon alpha and beta receptor subunit 2 |
| IL18 | interleukin 18 |
| IL1B | interleukin 1 beta |
| IL1R1 | interleukin 1 receptor type 1 |
| IL1R2 | interleukin 1 receptor type 2 |
| IRAK1 | interleukin 1 receptor associated kinase 1 |
| IRAK4 | interleukin 1 receptor associated kinase 4 |
| IRF1 | interferon regulatory factor 1 |
| KCNK6 | potassium two pore domain channel subfamily K member 6 |
| MAP3K5 | mitogen-activated protein kinase kinase kinase 5 |
| MAPK1 | mitogen-activated protein kinase 1 |
| MAPK3 | mitogen-activated protein kinase 3 |
| MAPK8 | mitogen-activated protein kinase 8 |
| MLKL | mixed lineage kinase domain like pseudokinase |
| MLST8 | MTOR associated protein, LST8 homolog |
| MYD88 | MYD88 innate immune signal transduction adaptor |
| NAIP | NLR family apoptosis inhibitory protein |
| Nek7 | NIMA related kinase 7 |
| NFKB1 | nuclear factor kappa B subunit 1 |
| NLRC4 | NLR family CARD domain containing 4 |
| NLRP1 | NLR family pyrin domain containing 1 |
| NLRP3 | NLR family pyrin domain containing 3 |
| NLRP6 | NLR family pyrin domain containing 6 |
| NLRP9 | NLR family pyrin domain containing 9 |
| NOD1 | nucleotide binding oligomerization domain containing 1 |
| NOD2 | nucleotide binding oligomerization domain containing 2 |
| P2RX7 | purinergic receptor P2X 7 |
| PKN1 | protein kinase N1 |
| POP1 | POP1 homolog, ribonuclease P/MRP subunit |
| PRKD1 | protein kinase D1 |
| PSTPIP1 | proline-serine-threonine phosphatase interacting protein 1 |
| PYCARD | PYD and CARD domain containing |
| PYDC2 | pyrin domain containing 2 |
| RHOA | ras homolog family member A |
| RIPK1 | receptor interacting serine/threonine kinase 1 |
| SCAF11 | SR-related CTD associated factor 11 |
| SHARPIN | SHANK associated RH domain interactor |
| SUGT1 | SGT1 homolog, MIS12 kinetochore complex assembly cochaperone |
| TICAM1 | toll like receptor adaptor molecule 1 |
| TIFA | TRAF interacting protein with forkhead associated domain |
| TLR4 | toll like receptor 4 |
| TNFRSF1A | TNF receptor superfamily member 1A |
| TRAF6 | TNF receptor associated factor 6 |
| TRIM11 | tripartite motif containing 11 |
| YWHAB | tyrosine 3-monooxygenase/tryptophan 5-monooxygenase activation protein beta |
