# Supplementary material for: A pyroptosis-related gene signature provides an alternative for predicting the prognosis of patients with hepatocellular carcinoma
Source: BMC Med Genomics. 2023 Jan 7;16:2. doi: 10.1186/s12920-023-01431-z (PMC9826587; doi:10.1186/s12920-023-01431-z)
Supplement: Supplementary file 2 — Additional file 2. Five kinds of immune infiltration algorithm principles. [file 12920_2023_1431_MOESM2_ESM.docx]

Table S2. Five kinds of immune infiltration algorithm principles

| Algorithm Name | Type | Comparisons | Algorithm | Cell types | Score |
| --- | --- | --- | --- | --- | --- |
| **CIBERSORT** | D | Intra | ν-support vector regression | 22 immune cell types | Immune cell fractions, relative to total immune cell content |
| **EPIC** | D | Intra, inter | constrained least square regression | 6 immune cell types, fibroblasts, endothelial cells | Cell fractions, relative to all cells in sample |
| **MCPCOUNTER** | M | Inter | mean of marker gene expression | 8 immune cell types, fibroblasts, endothelial cells | Arbitrary units, comparable between samples |
| **QUANTISEQ** | D | Intra, inter | constrained least square regression | 10 immune cell types | Cell fractions, relative to all cells in sample |
| **TIMER** | D | Inter | linear least square regression | 6 immune cell types | Arbitrary units, comparable between samples |
