# Supplementary material for: A pyroptosis-related gene signature provides an alternative for predicting the prognosis of patients with hepatocellular carcinoma
Source: BMC Med Genomics. 2023 Jan 7;16:2. doi: 10.1186/s12920-023-01431-z (PMC9826587; doi:10.1186/s12920-023-01431-z)
Supplement: Supplementary file 3 — Additional file 3. The siRNA sequences used in this study. [file 12920_2023_1431_MOESM3_ESM.docx]

| Table S3. siRNA | | |
| --- | --- | --- |
| gene | sense | anti-sense |
| CASP3 | UUUAAUGGAUUUUGAAUCCAC | GGAUUCAAAAUCCAUUAAAAA |
| IRAK1 | UGAUGUAGAAACUGAAUUGCC | CAAUUCAGUUUCUACAUCAGG |
| MAPK1 | UUGACAUUAUCAUAAGCAGAG | CUGCUUAUGAUAAUGUCAACA |
| MAPK3 | AAUCACAAAUCUUAAGGUCGC | GACCUUAAGAUUUGUGAUUUC |
| YWHAB | AGAUUUCUCUCUUCGUUGGAG | CCAACGAAGAGAGAAAUCUGC |
