# Supplementary material for: A pyroptosis-related gene signature provides an alternative for predicting the prognosis of patients with hepatocellular carcinoma
Source: BMC Med Genomics. 2023 Jan 7;16:2. doi: 10.1186/s12920-023-01431-z (PMC9826587; doi:10.1186/s12920-023-01431-z)
Supplement: Supplementary file 4 — Additional file 4. The primer sequences used in this study. [file 12920_2023_1431_MOESM4_ESM.docx]

| Table S4. primer sequences | | |
| --- | --- | --- |
| Primer | Forward sequence (5′ to 3′) | Reverse sequence (5′ to 3′) |
| HAVCR2 | GACTCTAGCAGACAGTGGGATC | GGTGGTAAGCATCCTTGGAAAGG |
| LGALS9 | GCAACACGAGGCAGAACGGAAG | GAAGCAGAGGTCAAAGGGCATCC |
| VTCN1 | TGACCAGGGAGCCAACTTCTCG | AGAGCACAGACACAACCTTCATGG |
| PDCD1 | AAGGCGCAGATCAAAGAGAGCC | CAACCACCAGGGTTTGGAACT |
| FGFR1 | GAGGCTACAAGGTCCGTTATG | GATGCTGCCGTACTCATTCT |
| PDGFB | CCATTCCCGAGGAGCTTTATG | GGTCATGTTCAGGTCCAACTC |
| PDGFRA | GGAAACAGAAACCGAGGTATGA | CTGCATCGGGTCCACATAAA |
| VEGFB | ATGATCCGGTACCCGAGCAGTC | GTCTGGCTTCACAGCACTGTCC |
