# Supplementary material for: A pyroptosis-related gene signature provides an alternative for predicting the prognosis of patients with hepatocellular carcinoma
Source: BMC Med Genomics. 2023 Jan 7;16:2. doi: 10.1186/s12920-023-01431-z (PMC9826587; doi:10.1186/s12920-023-01431-z)
Supplement: Supplementary file 5 — Additional file 5. Information of the antibody used in IHC. [file 12920_2023_1431_MOESM5_ESM.docx]

Table S5. Information of the antibody used in IHC.

| Gene | Antibody Name | Antibody Number | Company |
| --- | --- | --- | --- |
| CASP3 | Caspase 3/P17/P19 Monoclonal Antibody | 66470-2-Ig | Proteintech |
| IRAK1 | IRAK1 Polyclonal Antibody | 10478-2-AP | Proteintech |
| MAPK1/3 | ERK1/2 Polyclonal Antibody | 16443-1-AP | Proteintech |
| YWHAB | Anti-14-3-3 beta/alpha antibody | 0407-5 | HUABIO |
