# Supplementary material for: A pyroptosis-related gene signature provides an alternative for predicting the prognosis of patients with hepatocellular carcinoma
Source: BMC Med Genomics. 2023 Jan 7;16:2. doi: 10.1186/s12920-023-01431-z (PMC9826587; doi:10.1186/s12920-023-01431-z)
Supplement: Supplementary file 6 — Additional file 6. Figs. S1-S7. [file 12920_2023_1431_MOESM6_ESM.pdf]

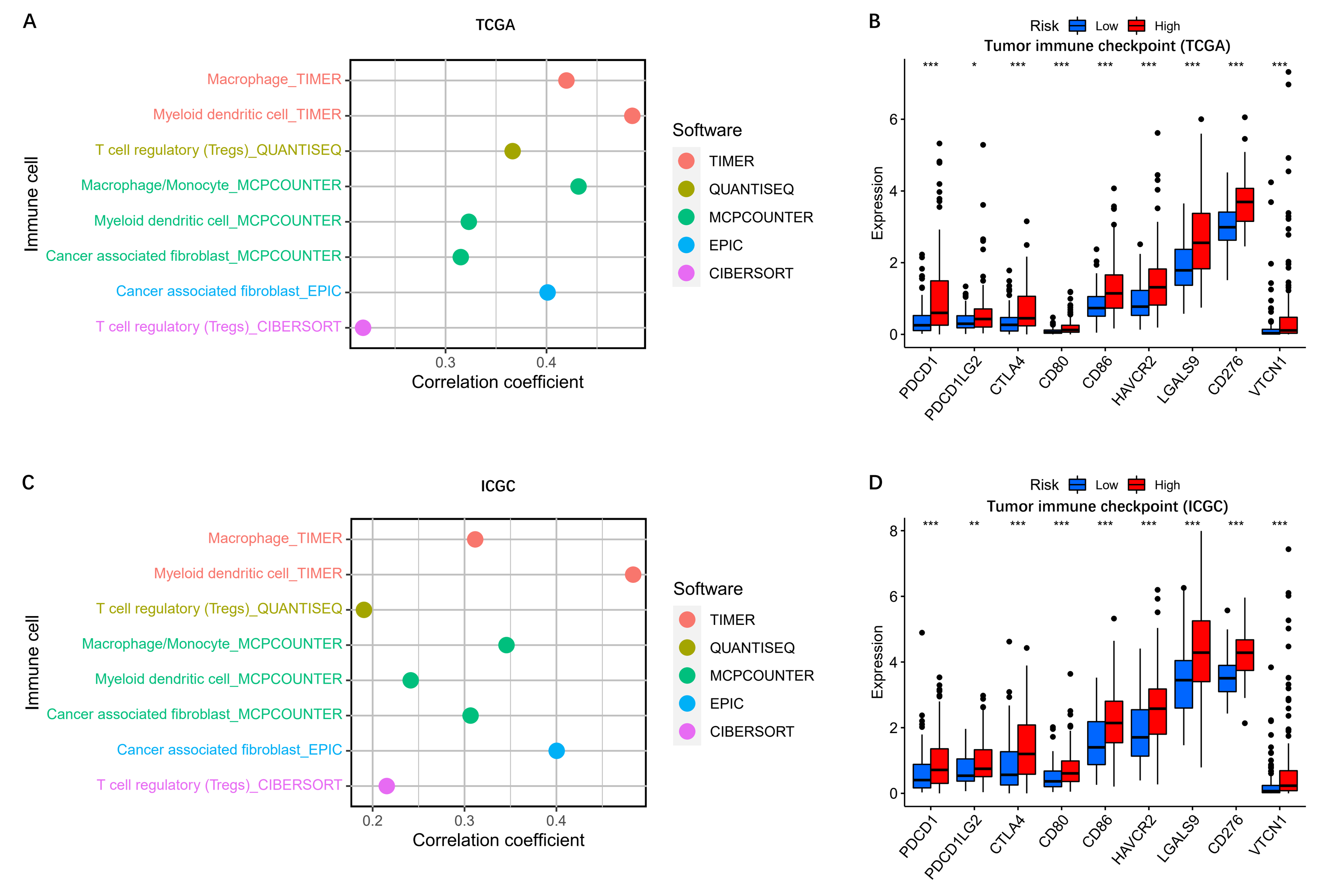

Fig. S1 Immune infiltrating cells and immune checkpoint genes analysis. TCGA cohort (A, B), ICGC cohort (C, D). (A, B) TIMER, QUANTISEQ, MCPCOUNTER, EPIC and CIBERSORT revealed the correlation between the risk score and tumor-infiltrating immune cells. (C, D) Immune checkpoint genes expression between different risk groups. P values are shown as ns, not significant; *, P < 0.05; **, P < 0.01; ***, P < 0.001.

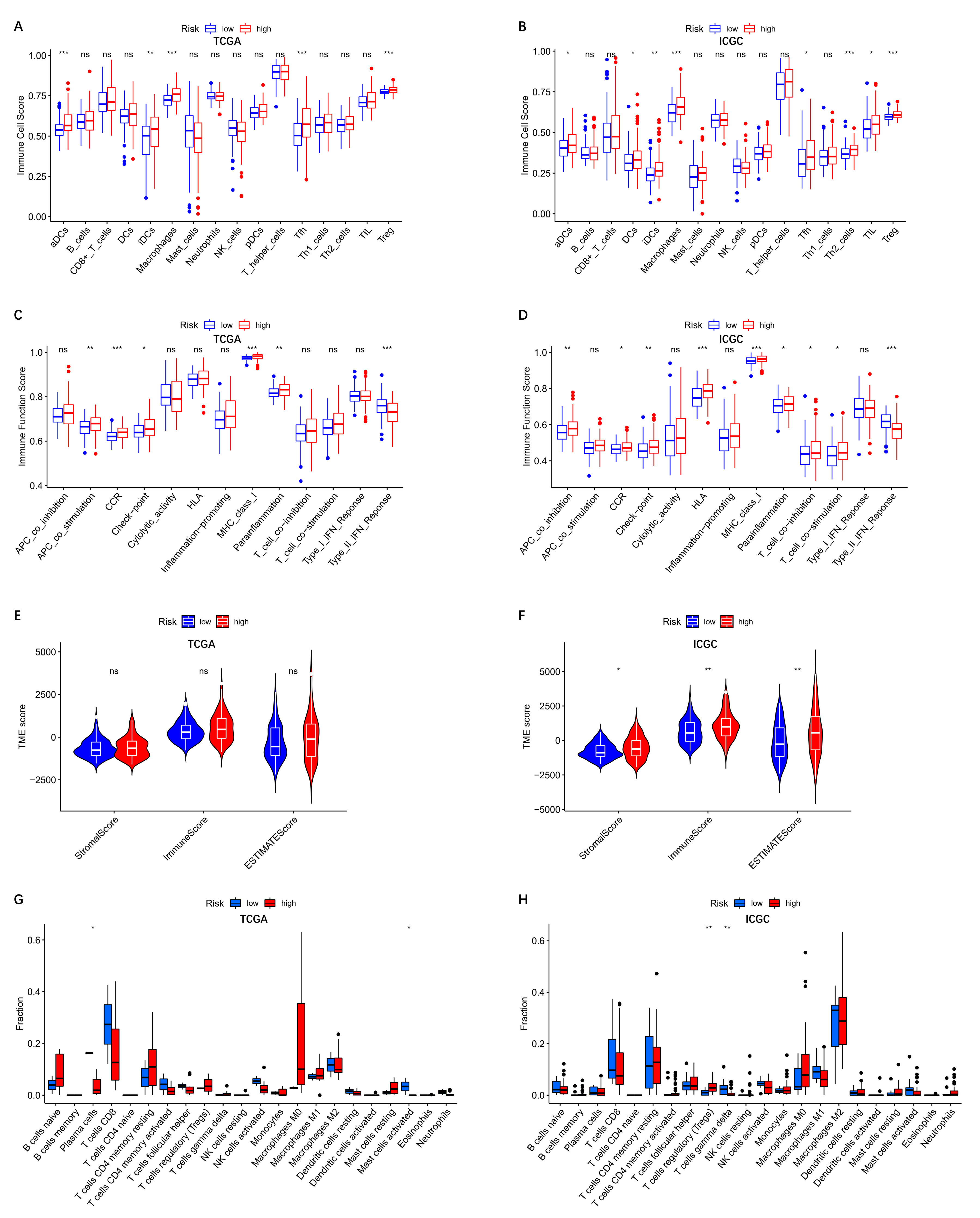

Fig. S2 Immune statuses between different risk groups. TCGA cohort (A, C, E, G), ICGC cohort (B, D, F, H). (A, B) The relative enrichment of 16 immune cells in different risk groups. (C, D) The relative enrichment of 13 immune-related functions in different risk groups. (E, F) StromalScore, ImmuneScore and ESTIMATEScore in two subgroups. (G, H) The proportion of the 22 immune cells detected by the CIBERSORT algorithm in different risk groups. P values are shown as ns, not significant; *, P < 0.05; **, P < 0.01; ***, P < 0.001.

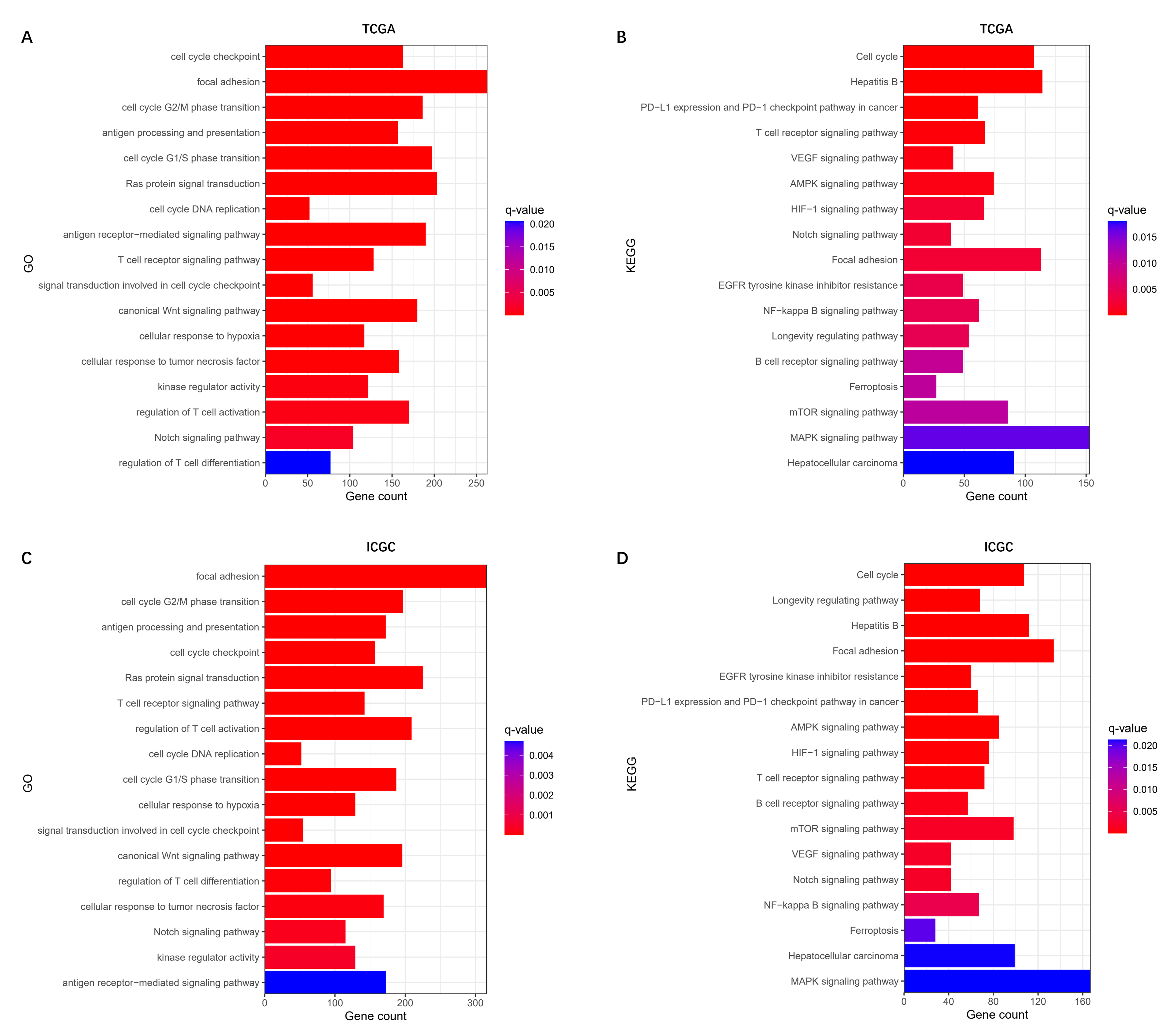

Fig. S3 Enrichment analysis of biological function and pathway. TCGA cohort (A, B), ICGC cohort (C, D). (A, C) GO, Gene Ontology. (B, D) KEGG, Kyoto Encyclopedia of Genes and Genomes.

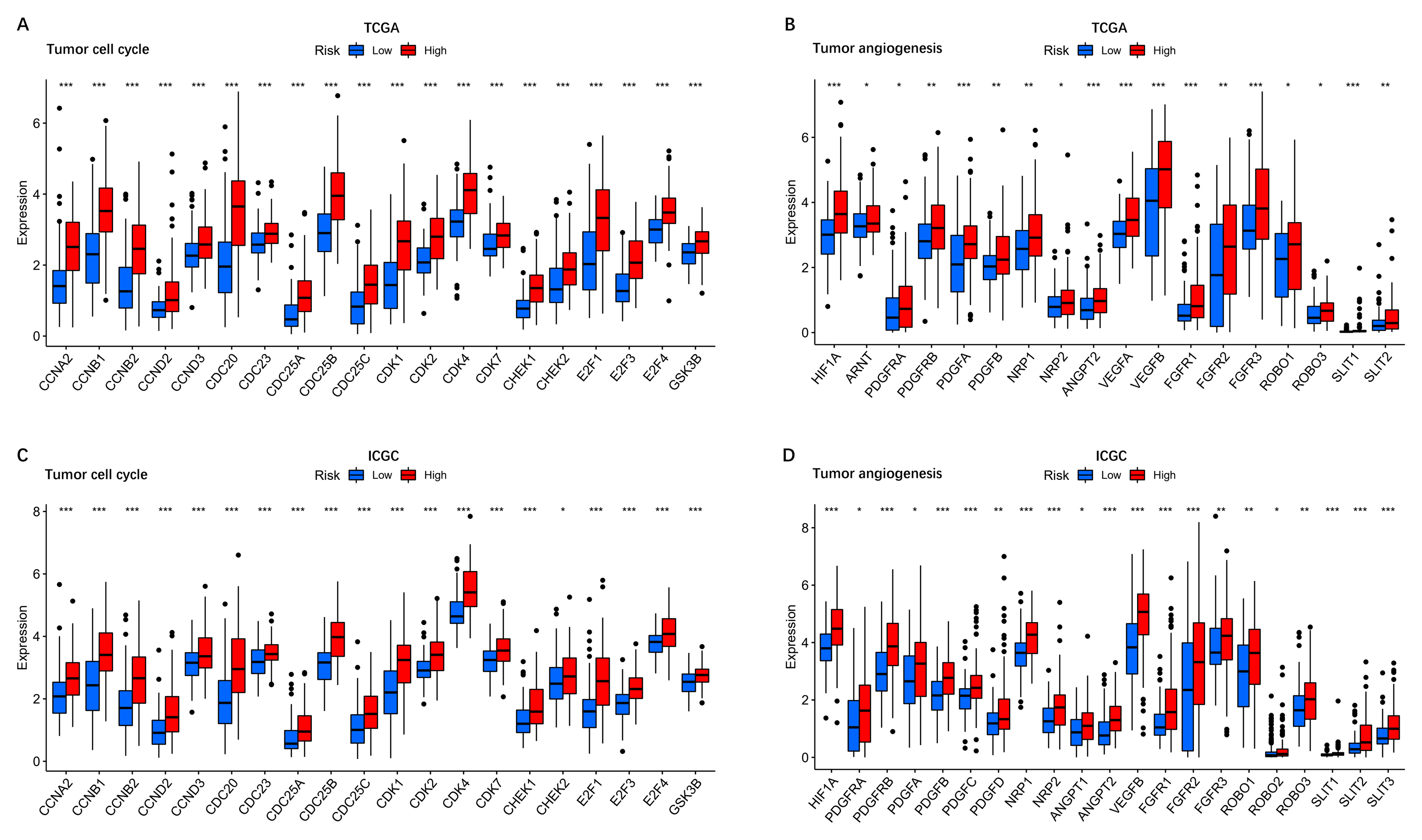

Fig. S4 Cell cycle and tumor angiogenesis genes expression between different risk groups. TCGA cohort (A, B), ICGC cohort (C, D). (A, C) Cell cycle gene expression between different risk groups. (B, D) tumor angiogenesis genes expression between different risk groups. P values are shown as ns, not significant; *, P < 0.05; **, P < 0.01; ***, P < 0.001.

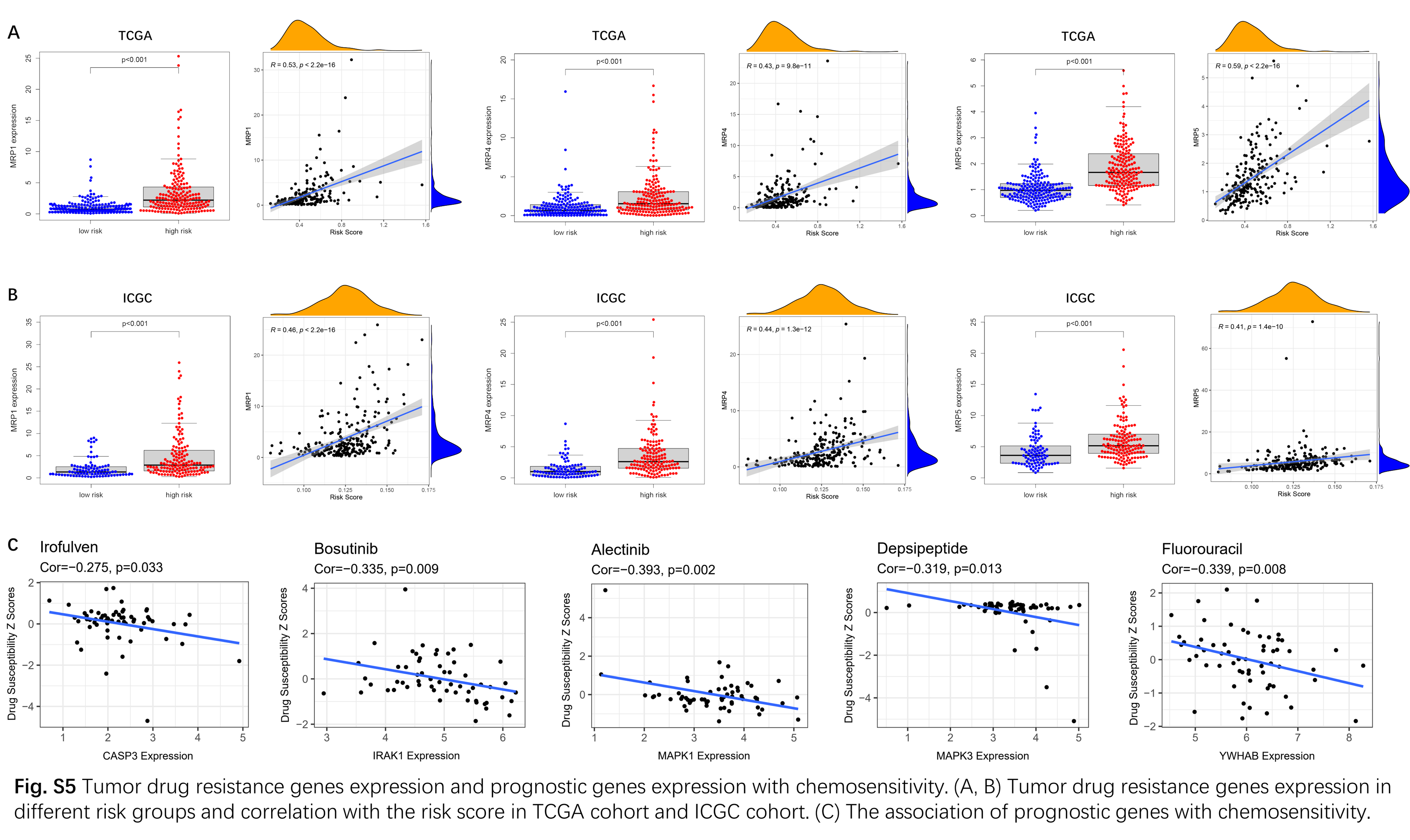

Fig. S5 Tumor drug resistance genes expression and prognostic genes expression with chemosensitivity. (A, B) Tumor drug resistance genes expression in different risk groups and correlation with the risk score in TCGA cohort and ICGC cohort. (C) The association of prognostic genes with chemosensitivity.

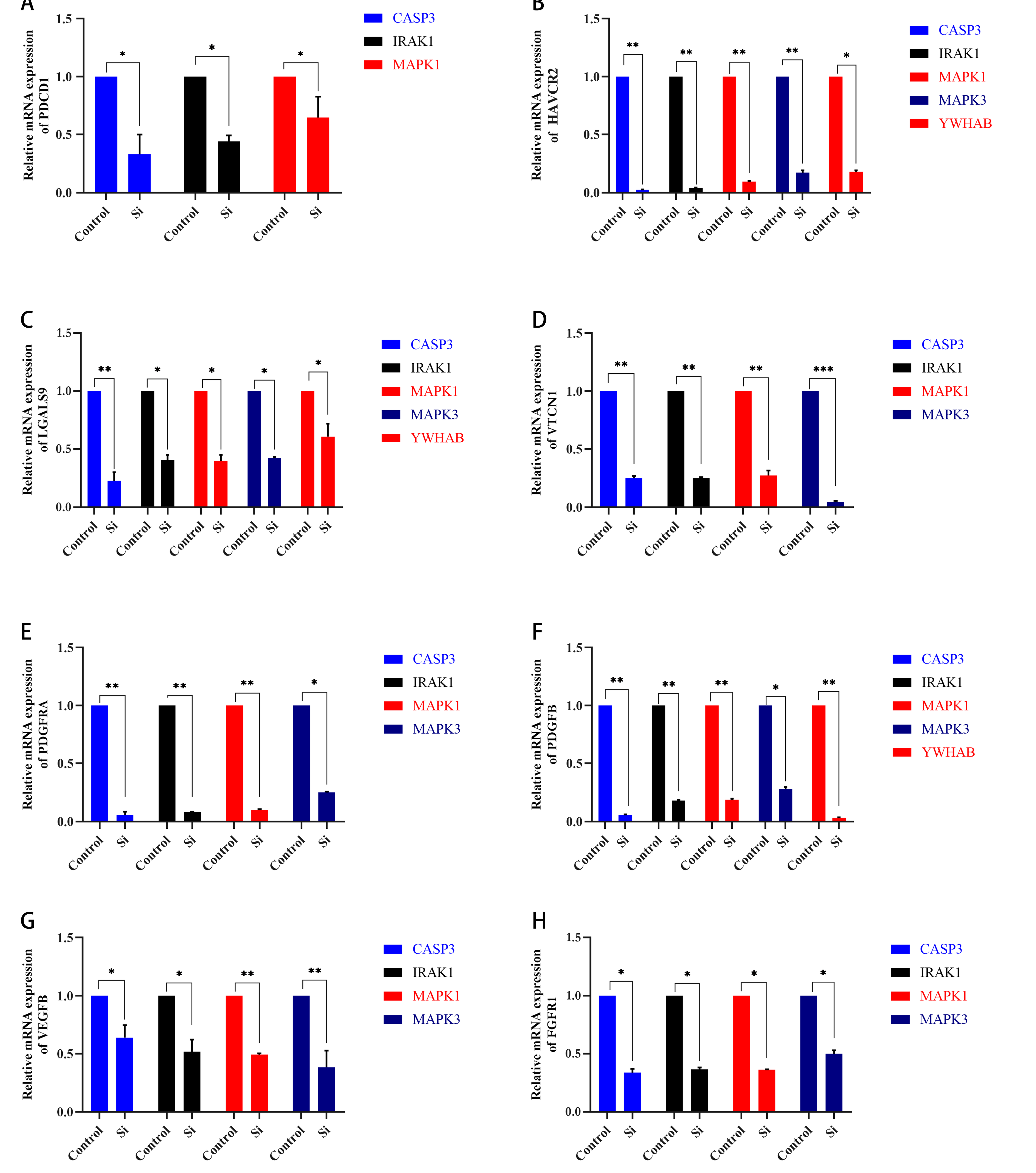

Fig. S6 The mRNA expression changes of immune checkpoint genes and angiogenesis-related genes in HCC cells. (A-D) Immune checkpoint genes: PDCD1, HAVCR2, LGALS9 and VTCN1. (E-H) Angiogenesis-related genes: PDGFRA, PDGFB, VEGFB and FGFR1.

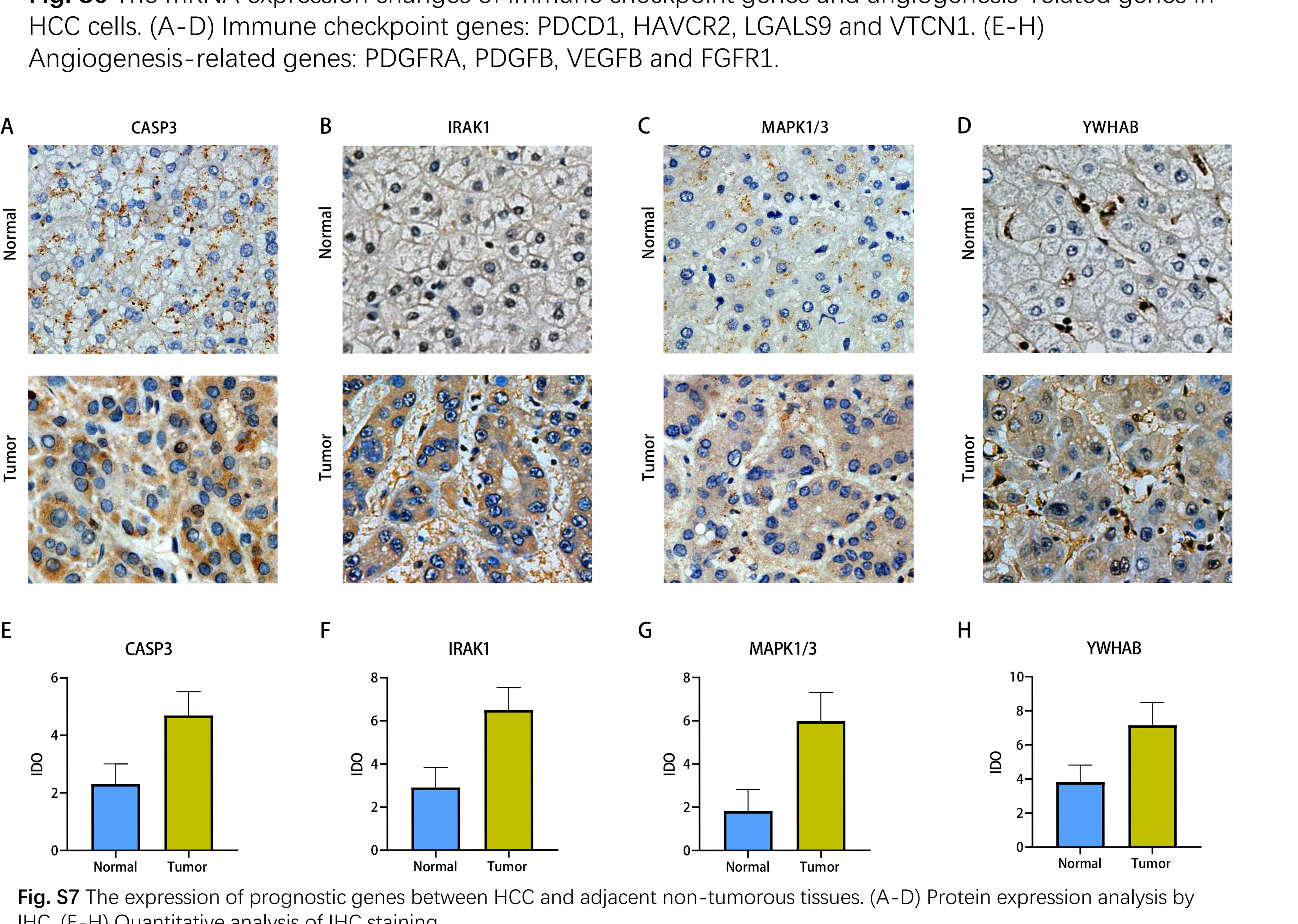

Fig. S7 The expression of prognostic genes between HCC and adjacent non-tumorous tissues. (A-D) Protein expression analysis by IHC. (E-H) Quantitative analysis of IHC staining.
